# Supplementary material for: TFvelo: gene regulation inspired RNA velocity estimation
Source: Nat Commun. 2024 Feb 15;15:1387. doi: 10.1038/s41467-024-45661-w (PMC11258302; doi:10.1038/s41467-024-45661-w)
Supplement: Supplementary file 1 — Supplementary Information [file 41467_2024_45661_MOESM1_ESM.pdf]

Supplementary Information

Table of contents

1. Estimating the current RNA velocity as a linear combination of TFs' expressions. 2
2. The clockwise curve on the joint distribution plot between expressions levels of a TF-target pair. 5
3. Details in the experiment on synthetic dataset 6
4. Additional results on Pancreas dataset. 9
5. Additional results on gastrulation erythroid dataset. 21
6. The root and end cells detection. 24
7. All TFs list 25
8. Supplementary References 30

1. Estimating the current RNA velocity as a linear combination of TFs' expressions.

The RNA velocity modeled by scVelo of a target gene can be approximately represented as a linear combination of TFs' expression level. Given a target gene, we use Least Absolute Shrinkage and Selection Operator (LASSO) regression to predict its RNA velocity (modeled by scvelo) based on the total mRNA abundance of TFs and the target gene itself (**Fig. S1a**). The input feature includes the preprocessed total mRNA abundance of all candidate TFs and the target gene itself. RNA velocity modeled by scVelo of the target gene is chosen as the label. An additional normalization is adopted to ensure each dimension in the input being satisfied a normal distribution.
LASSO is a linear regression method that combines the variable selection with an L1 regularization to enhance the prediction accuracy as well as the sparsity. For a target gene g, suppose S_g represent the set of those TFs whose corresponding weight is non-zero, the velocity of gene g can be written as

$$v_g = \sum_{TF_i \in S_g} w_{g,TF_i} e_{TF_i} + w_{g,g} e_g \quad (1)$$

where e_{TF_i} and e_g are the expression level of TF_i and gene g respectively, w_{g,TF_i} and $w_{g,g}$ are the weights.

On the scRNA-Seq pancreas dataset, we first preprocess the data using the scVelo's tutorial, where the preprocessed pancreas dataset comprises 3,696 cells and 2,000 genes, and identify 143 TFs according to the annotation [1]. We only run the LASSO model on those genes that defined as velocity genes by scVelo, which means that scVelo is regard to be able to fit the dynamics of these genes, where an additional target gene filtering is that the smoothed expression needs to be non-zero in over 3,000 cells. The training set comprises 80% of the cells, while the remaining 20% are allocated to the testing set. As shown in **Fig. S1b**, the linear model can achieve low testing loss on some genes. Visualization of the regression results on the testing set for each target gene indicates a high correlation between the predicted and labeled velocity, as demonstrated in **Fig. S1c**. By utilizing the sparse model learned by LASSO, velocity modeled by scVelo can be approximately represent as a linear combination of some of TFs. This model suggests that changes in the target gene are strongly associated with the expressions of selected TFs.

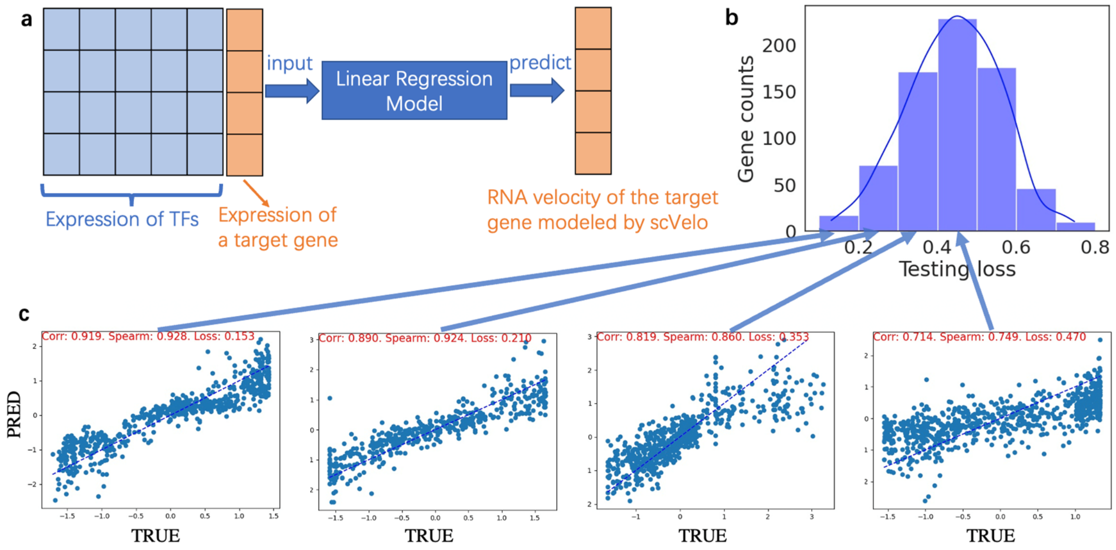

Figure S1. Estimating the RNA velocity modeled by scVelo as a linear combination of TFs' expressions. (a) The workflow of linear regression. Cells are divided in to training and testing sets, and LASSO model is employed for linear regression. (b) The distribution of testing loss. (c) The plot between the predicted RNA velocity by LASSO and true labels on the test set for 4 example genes, which are DGKB, ABCC8, ACTN4 and AFF2, respectively. The dot line refers to True=Predict. The correlation coefficient, spearman correlation and MSE loss on test set are texted on the figure.

Then, we further analyzed TF-target pairs with positive weights in LASSO models and validated them against ENCODE TF-target datasets. These datasets label the regulatory relationship between a TF and a target gene based on the binding of the transcription factor near the transcription start site of genes. On these TFs detected on the pancreas dataset, we could find the annotation on 21 TFs of them from the ENCODE TF-target database. For each of the 21 TFs analyzed, we calculate the ratio of TF-target pairs that can be validated with a regulatory relationship. To test the statistical significance, a background is determined by the probability of a randomly picked gene being regulated by each TF. our results (**Table S1**) indicate that TF-target pairs associated with non-zero weights show the higher validation ratio on most TFs. Using a one-sided t-test, we found that TF-target pairs with positive weights were significantly more likely to be functionally related to the target gene (n=21 TFs, p=6.66e-06).

Table S1. The ratio of TF-target pairs that can be verified by ENCODE dataset.

TF	Background			TFs with non-zero weights		
	Verified	Total	Ratio	Verified	Total	Ratio
**TCF7L2**	1275	2000	0.638	184	253	**0.727**
**CREB1**	1446	2000	0.723	359	459	**0.782**

**KDM5B**	1411	2000	0.706	264	348	**0.759**
**FOXP2**	1206	2000	0.603	293	449	**0.653**
**EGR1**	1364	2000	0.682	290	398	**0.729**
**REST**	1649	2000	0.825	252	290	**0.869**
**FOS**	1249	2000	0.625	286	428	**0.668**
**EBF1**	1232	2000	0.616	339	518	**0.654**
**ARID3A**	1224	2000	0.612	274	423	**0.648**
**E2F1**	365	2000	0.183	110	510	**0.216**
**USF2**	1436	2000	0.718	214	287	**0.746**
**HNF4A**	414	2000	0.207	99	429	**0.231**
**NR3C1**	441	2000	0.221	90	370	**0.243**
**STAT3**	1336	2000	0.668	252	370	**0.681**
**MXI1**	1725	2000	0.863	365	417	**0.875**
**MEF2A**	305	2000	0.153	63	385	**0.164**
**HMGN3**	1360	2000	0.680	176	255	**0.690**
**ATF3**	827	2000	0.414	181	430	**0.421**
**SREBF2**	29	2000	0.015	8	526	0.015
**GTF2B**	672	2000	0.336	89	265	0.336
**ZEB1**	878	2000	**0.439**	215	496	0.433

2. The clockwise curve on the joint distribution plot between expressions levels of a TF-target pair.

As shown in **Fig. S2**, the clockwise curve can be seen on the joint distribution of the abundance between some TF-target pairs, which means the phase delay between them and future indicate the potential regulatory relationship between them. This finding also motivates us to explore the gene dynamics based on the phase delay between TF and target.

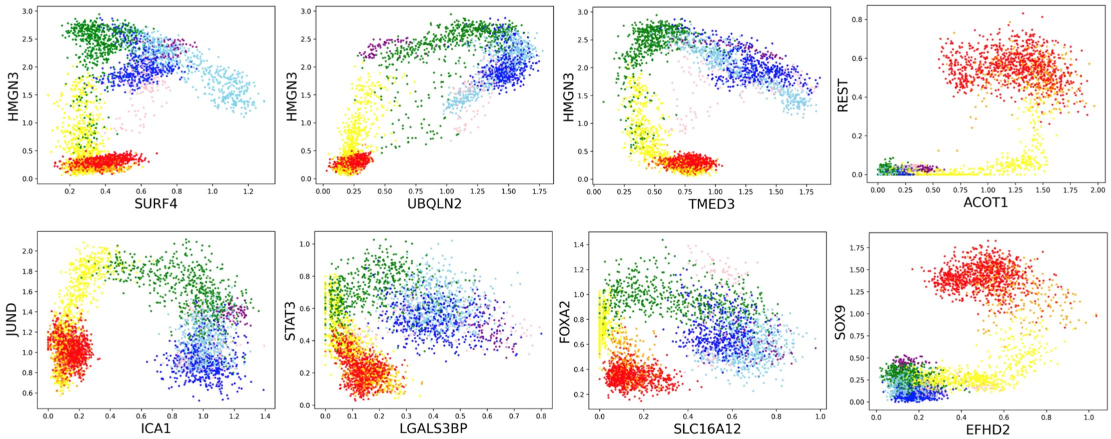

Figure S2. The joint distribution between the abundance of TF-target pairs on pancreas dataset. Cells are colored in the same way as that in **Fig. S4b**.

3. Details in the experiment on synthetic dataset

To evaluate the proposed computational framework in TFvelo, we verify it with a synthetic dataset firstly. In the synthetic dataset, the dynamic model of a target gene is designed to have the same formulation with that in the TFvelo model, which is

$$y = \alpha\, sin(2\pi t + \theta) + \beta,$$

$$\frac{dy}{dt} = \mathbf{WX} - \gamma y\,.$$

To generate the ground truth dynamics satisfying these constraints, we firstly sample the value for parameter $\alpha, \beta, \theta,\ \gamma$, the weight vector W and assign the cell-specific latent time t, randomly. Then the WX and y can be calculated according to dynamic functions. After that, X can be generated analytically based on the $\mathbf{WX}$ and $\mathbf{W}$. Finally, random noise is added to both $\mathbf{X}$ and y. X and y will be input to TFvelo, while all parameters $[\alpha, \beta, \theta,\ \gamma]$, weights $\mathbf{W}$ and the cell-specific latent time t need to be inferred. The TFvelo will reconstruct the dynamic function and learn the weight of each TF. In the synthetic dataset, a target gene is regulated by 10 TFs, where genes are from 1000 cells. The corresponding weights can be either positive or negative, which reflect to TFs that may up-regulate and down-regulate the target gene, respectively.

We take experiment on 200 randomly generated dynamics. The performance of TFvelo was evaluated using the spearman correlation between the ground truth and the reconstructed values. Under 20 iterations for optimization, the spearman correlation coefficient between the ground truth weights and inferred weights of TFs is 0.823, and that between the ground truth velocities and inferred velocities is 0.894 (**Fig. 1.x**). The high consistence shows that TFvelo can effectively reconstruct the underlying dynamics. For further analyzing the learned weights corresponding to each TF, The F1 score can be obtained according to the flowing definition of confusion matrix.

Table S2. The confusion matrix defined on synthetic dataset. The counts under being optimized by 20 iterations are shown.

	Positive weight in reconstruction	Negative weight in reconstruction
Positive weight in ground truth	*TP* (1475)	*FN* (144)
Negative weight in ground truth	*FP* (31)	*TN* (350)

$$Precision = \frac{TP}{TP + FP}$$

$$Recall = \frac{TP}{TP + FN}$$

$$F1\ score = \frac{2 * Precision * Recall}{Precision + Recall}$$

Then we label those TFs with positive ground truth weights as 1, and those TFs with negative ground truth weights as 0. After that, the receiver operating characteristic (ROC) curve based on the learned weights of the TFs can be drawn.

The high F1 score (0.944) and high ratio of area under the receiver operating characteristic (ROC) curve (0.962) also demonstrate that TFvelo can correctly recognize whether the weight is positive or negative. These results suggest that the ground truth weights and learned weights are consistent, indicating that the computational framework of TFvelo can effectively reconstruct the system dynamic and detect potential gene regulation relationships. Therefore, we conclude that the computational framework of TFvelo is a reliable method for detecting gene regulation relationships and reconstructing system dynamics.

The performance under different number of iterations is explored in **Fig. S3**.

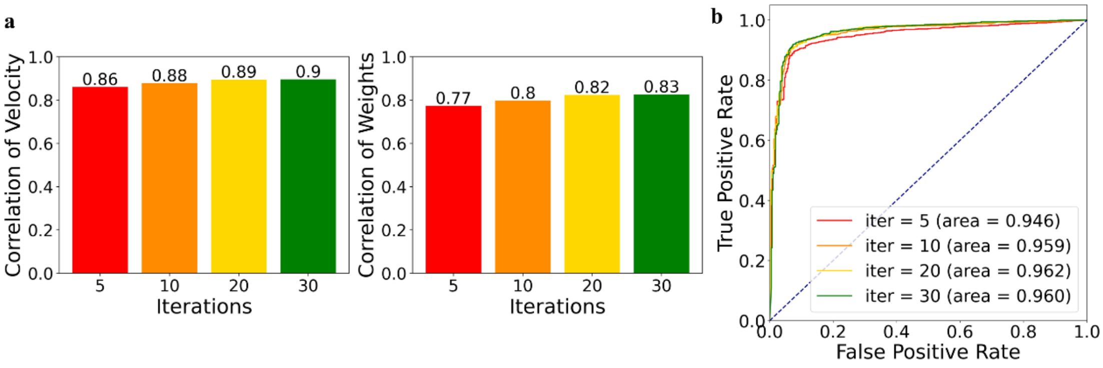

Figure S3. The comparison of performance under different number of iterations. (a) Spearman correlation coefficient between ground truth velocity/weights and inferred velocity/weights on all synthetic data. (b) The AUROC.

On the synthetic data, for each cell i, we can observe $x_i \in R^{n\_gene}$ and $y \in R$. There were no baseline approaches in RNA velocity or trajectory inference trying to fit this data. To add a baseline comparison, we employ a vanilla EM approach as baseline here, by removing the optimization step on weight, as well as the strategy of optimizing from multiple initial points. After optimizing for 20 iterations in common, the performance of this baseline is much poorer than TFvelo (**Fig. S4**). The inferred velocity is weakly correlated with the ground truth velocity (**Fig. S4a,b**). **Fig. S4c** shows three examples, where the EM baseline can not extract the dynamics Due to its inability to optimize data distribution on the phase portrait or escape local minima.

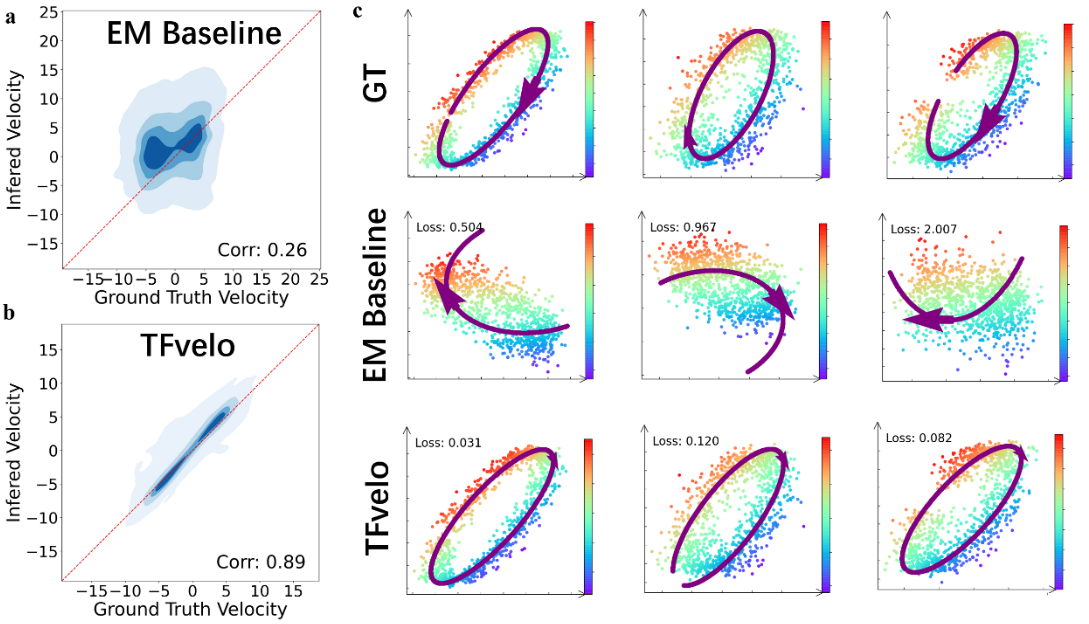

Figure S4. The performance of the simple EM baseline on synthetic dataset. (a) The joint distribution between the ground truth velocity and inferred velocity by EM the baseline. The spearman correlation between them is printed. (b) The joint distribution between the ground truth velocity and inferred velocity by TFvelo approach. The spearman correlation between them is printed. (c) Three examples for illustrating the comparison between the dynamics fittings of the baseline and TFvelo. Source data are provided in the Supplementary Data file.

4. Additional results on Pancreas dataset.

The results obtained by multiple RNA velocity approaches and pseudotime inference approaches are shown in **Fig. S5**. To assess the gene-specific latent time directly, we directly pool the learned $t_{g,c}$ over all genes without applying any smoothing to get the t_c^{latent} for cell c:

$$t_c^{latent} = \frac{1}{n_{gene}} \sum_{g=1}^{n_{gene}} t_{g,c}$$

The results about this latent time t^{latent} after being normalized to 0 to 1 are shown in **Fig. R5.** The spearman correlation between the Palantir pseudotime and the mean latent time obtained with all genes is 0.788.

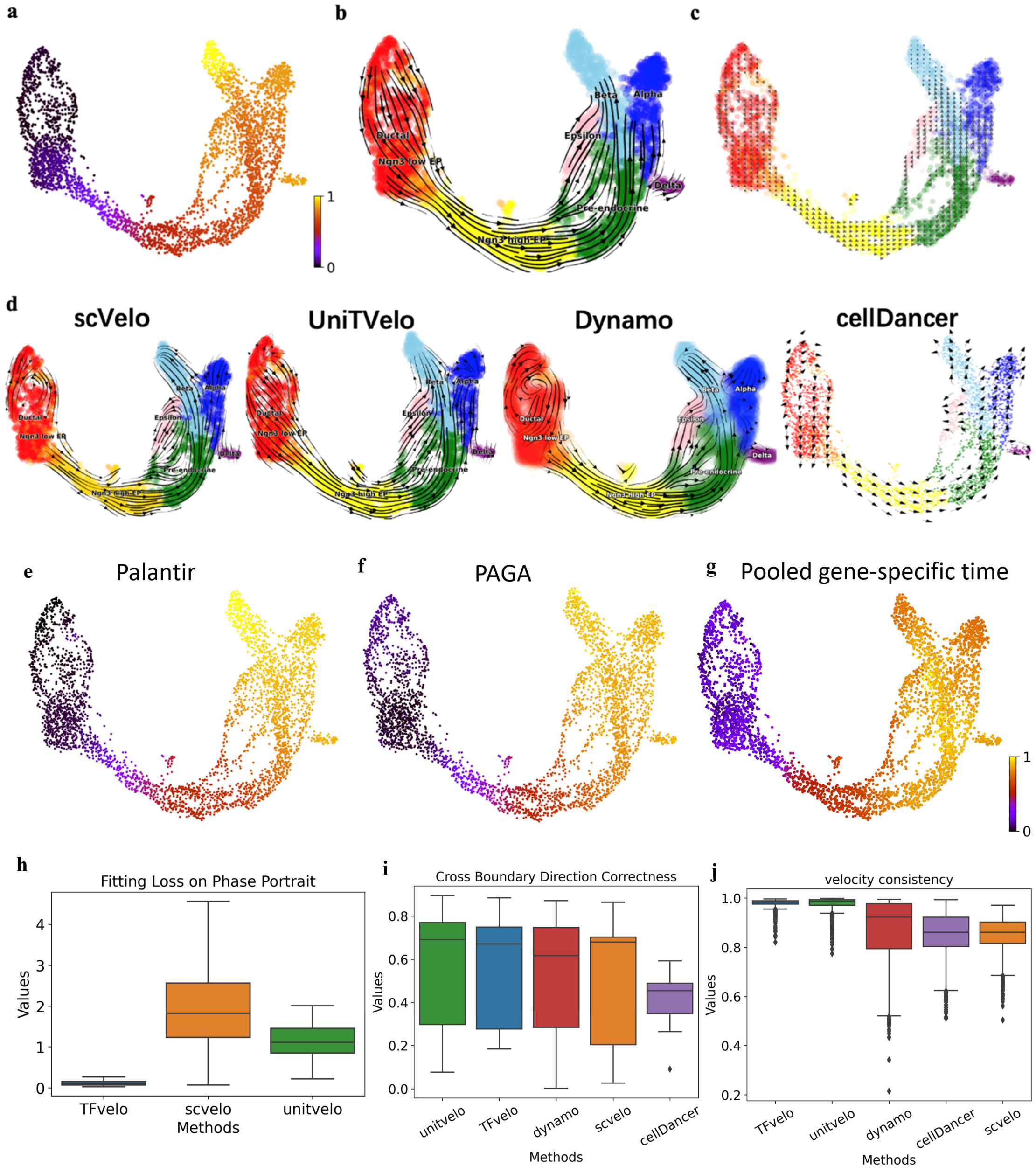

Figure S5. Comparison between TFvelo with baseline approaches on pancreas dataset**.** (a) Pseudotime inferred by TFvelo on UMAP space. (b) Stream plot of TFvelo on UMAP space. (c) Grid plot of TFvelo on UMAP space. (d) Stream plot of baseline RNA velocity approaches on UMAP space, where the results are obtained by run the pipeline of them. (e) Pseudotime inferred by Palantir on UMAP space. (f) Pseudotime inferred by PAGA on UMAP space. (g) The mean gene-specific time learned by TFvelo. Pannel e, f and g share the same color bar. (h) Comparison on the phase portrait fitting loss. (i) Comparison on cross-boundary direction correctness (j) Comparison on velocity consistency. Source data are provided in the Supplementary Data file.

The sparsity in data is a common challenge for scRNA-seq studies. Considering that only about 20% of reads contained unspliced intronic sequences[2], the sparse unspliced abundance is a main obstacle for dynamics fitting in phase portrait. Our quantitative analysis of the sparsity in unspliced, spliced and total mRNA counts on pancreas dataset is shown in **Fig. S6a**, which verifies the high sparsity in unspliced counts. **Fig. S6b** shows the comparison on two genes with sparse unspliced counts to illustrate the advantage of TFvelo for addressing the issue of sparsity. Although these genes can pass the filtering and selection during preprocessing, they are still too sparse to provide sufficient information for fitting a model well.

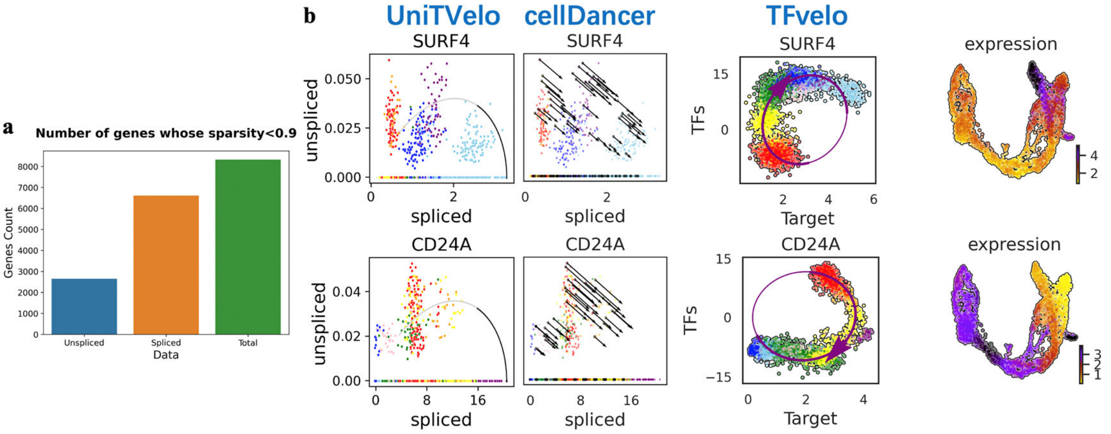

Figure S6. The sparsity analysis on pancreas dataset. (a) The comparison of sparsity between unspliced, spliced and total mRNA counts. The sparsity of a gene is defined as: $\text{Sparsity} = \frac{\text{The number of cells that the count of this gene is zero}}{\text{The total number of cells}}$. (b) The phase portrait fitting of each method on two genes with sparse unspliced counts, scVelo or Dynamo is not shown because they do not construct dynamics on these genes.

In the main manuscript, we show the phase portrait fitting on gene LITAF (**Fig. 1a**), H19 and MAML3 (**Fig. 2c**). Here, we compare the gene-specific latent time learned by scVelo and TFvelo on these genes in **Fig. S7**.

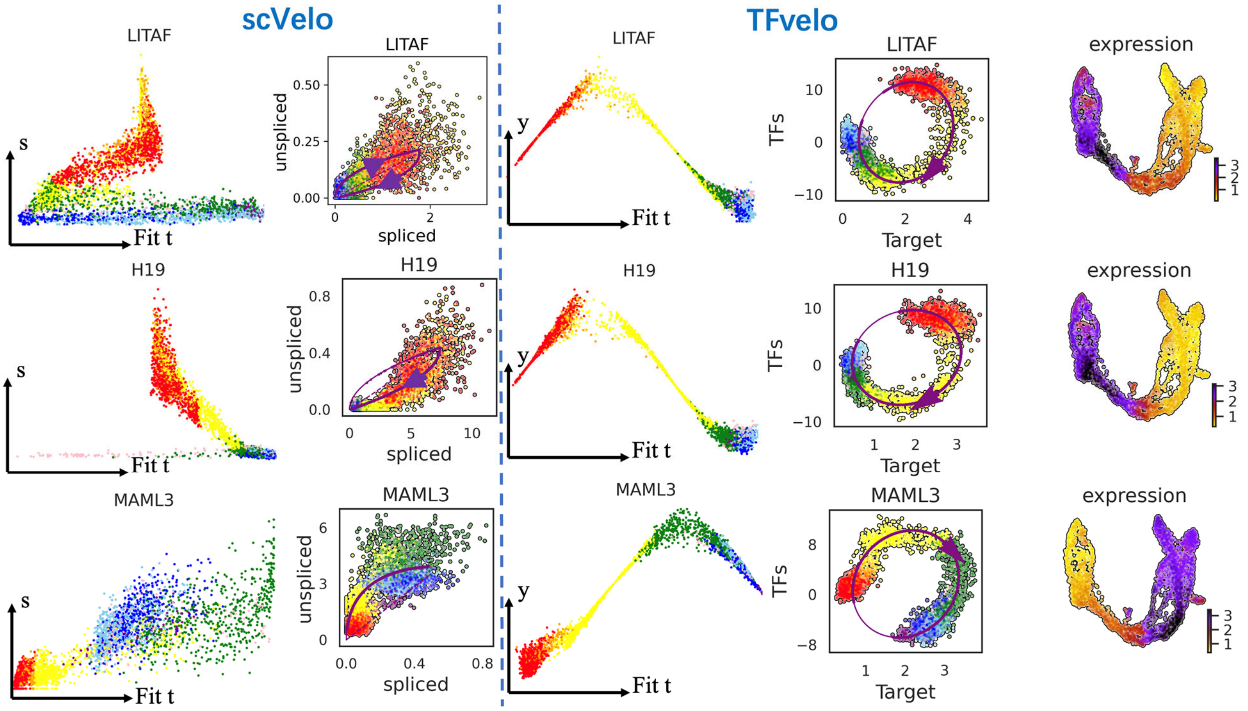

Figure S7. Comparison between scVelo and TFvelo on gene LITAF, H19 and MAML3. From left to right on each row: Gene expression dynamics resolved along gene-specific latent time obtained by scVelo, the phase portrait fitting obtained by scVelo, gene expression dynamics resolved along gene-specific latent time obtained by TFvelo, the phase portrait fitting obtained by TFvelo, and the expression level on UMAP. Cells are colored in the same way as **Fig. S5b**.

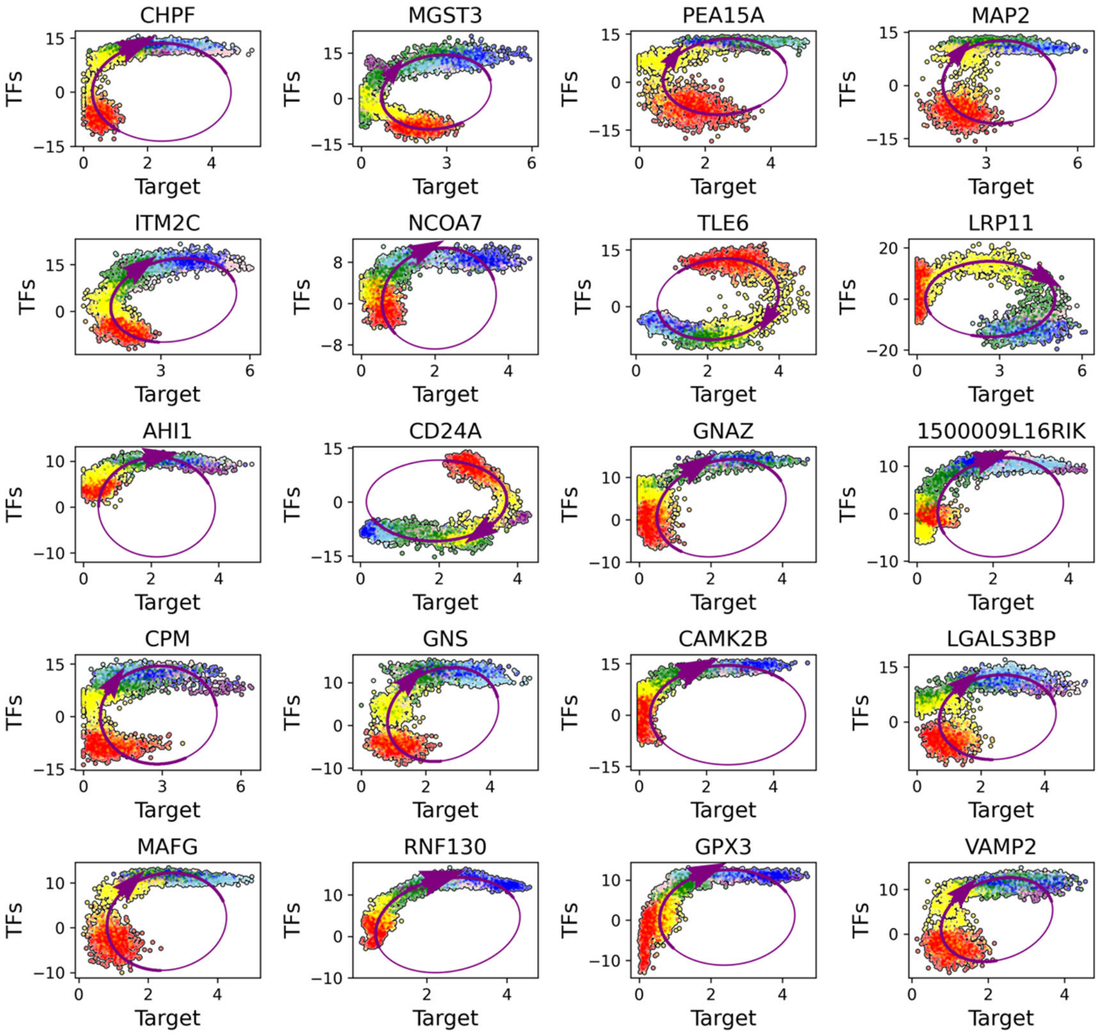

Figure S8. The phase portrait fitting of 20 example genes from pancreas dataset. Cells are colored in the same way as **Fig. S5b**.

We also systematically evaluate TFvelo using a series of genes that may play crucial roles in the pancreas differentiation process. We selected genes according to the paper that proposed the pancreas dataset [3]. From the section of "single cell RNA-seq of the embryonic EP-enriched pancreatic epithelial cells" in that paper, 33 genes were introduced as marker genes in the pancreas differentiation process, which are DLK1, CPA1, MYC, NOTCH2, PTF1A, CEL, RBPJL, SOX9, ANXA2, BICC1, NGN3, HES6, FEV, CCK, NEUROD1, RBP4, PYY, CHGB, MDK, BTF3, VTN, JAM3, CBX3, HMGN1, YBX1, REEP5, SPP1, BTBD17, GADD45A, VWA5B2, TOX3, TMEM97, FAM183B, CBFA2T3, RCOR2, SMARCD2, INSM1, CBFA2T2, SPP1, TMSB10, MDK, MARCKSL1, CDK4, and SOX4. Among them, 22 genes are selected by the pre-processed step of both TFvelo and scVelo, which are FAM183B, RBP4, SPP1, SOX9, GADD45A, MDK, TMEM97, DLK1, VWA5B2, ANXA2, BICC1, PYY, FEV, CCK, CHGB, CPA, BTBD17, JAM3, NEUROD1, NOTCH2, RBPJL and TOX3.

We next compare the fitting by TFvelo and scVelo on these 22 genes in **Figs. S9-11**, which is quantitively evaluated by the Spearman correlation between these gene-specific latent time and Palantir pseudotime (**Fig. S5e** and **Fig. S12**). As shown in the phase portrait fitting of all the 22 genes in **Figs. S9-11,** scVelo fails to fit 10 genes whose Spearman correlation is set as 0. In contrast, TFvelo gets high spearman correlation (larger than 0.6) for 11 genes, and a much higher median value (0.58) compared to scVelo (0.00), indicating that TFvelo can provide a much more accurate fitting for these important genes.

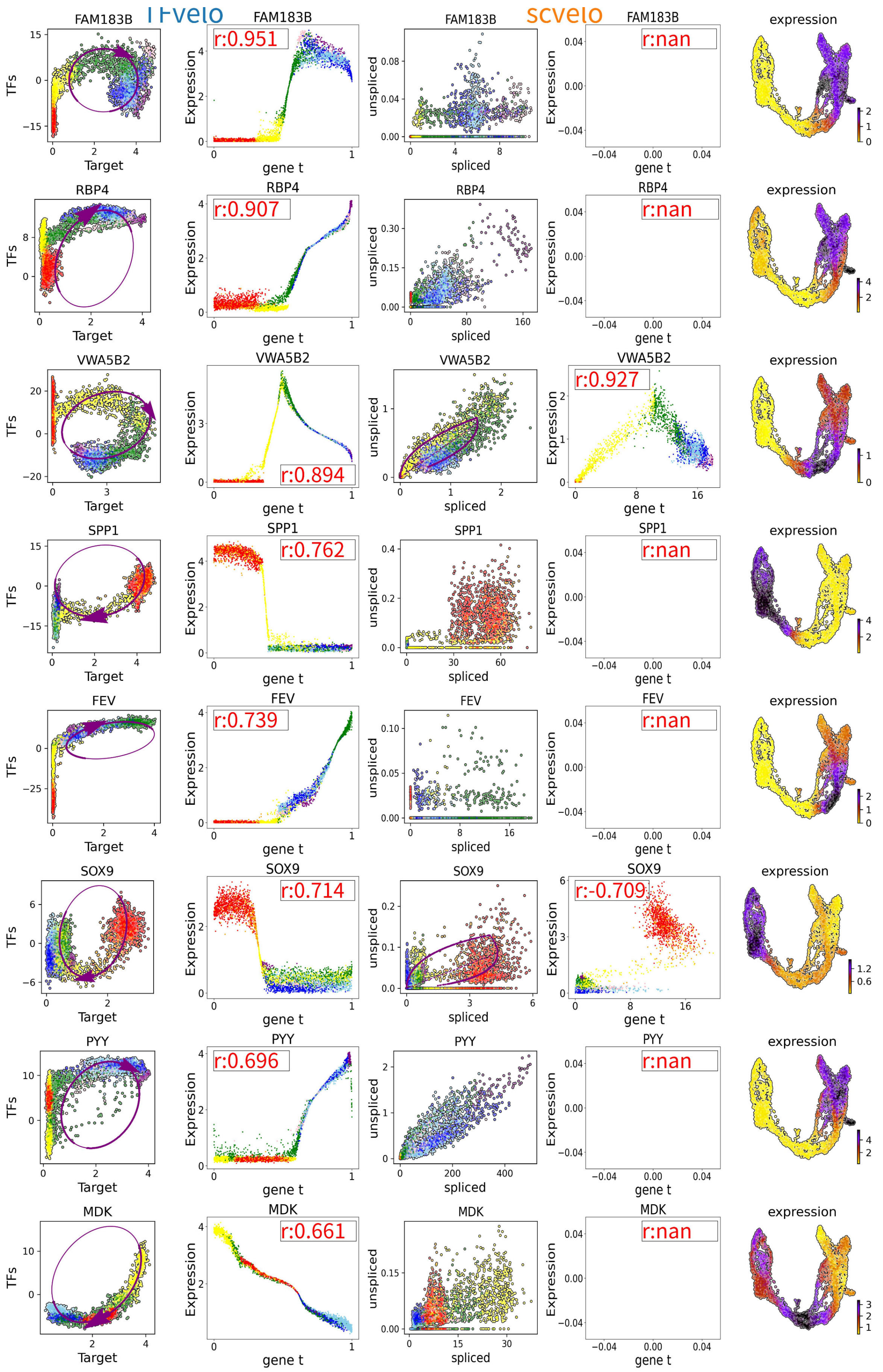

Figure S9. The comparison between TFvelo and scvelo on the genes selected from the pancreas study, including the dynamics fitting in phase portrait (1st and 3rd columns

for TFvelo and scvelo respectively), and the expression along gene-specific latent time (2nd and 4th columns for TFvelo and scvelo respectively). The spearman correlation between each gene-specific time with Palantir pseudotime is shown in the plot.

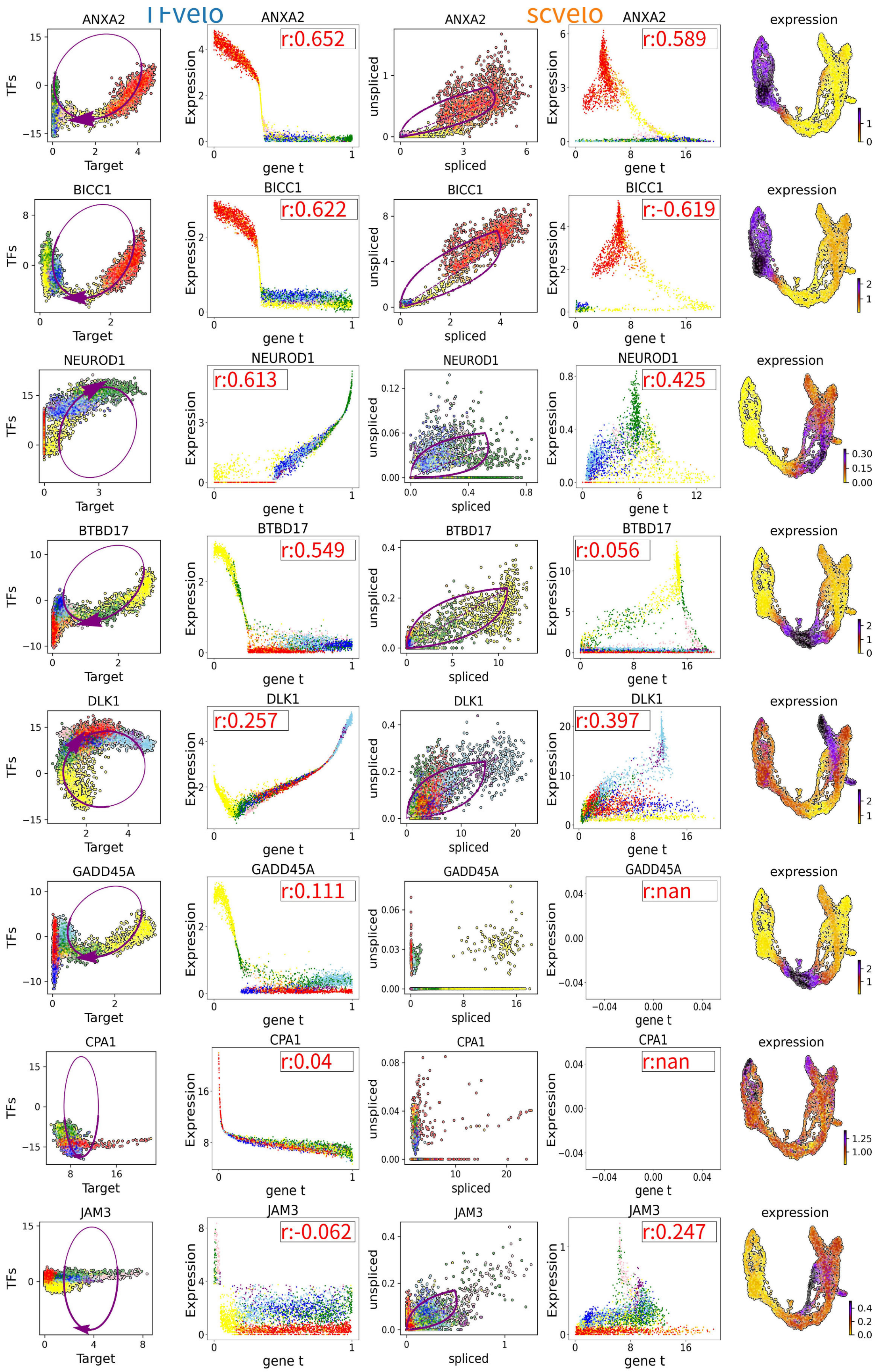

244

245 **Figure S10.** The comparison between TFvelo and scvelo on the genes selected from
246 the pancreas study, including the dynamics fitting in phase portrait (1st and 3rd columns

for TFvelo and scvelo respectively), and the expression along gene-specific latent time (2nd and 4th columns for TFvelo and scvelo respectively). The spearman correlation between each gene-specific time with Palantir pseudotime is shown in the plot.

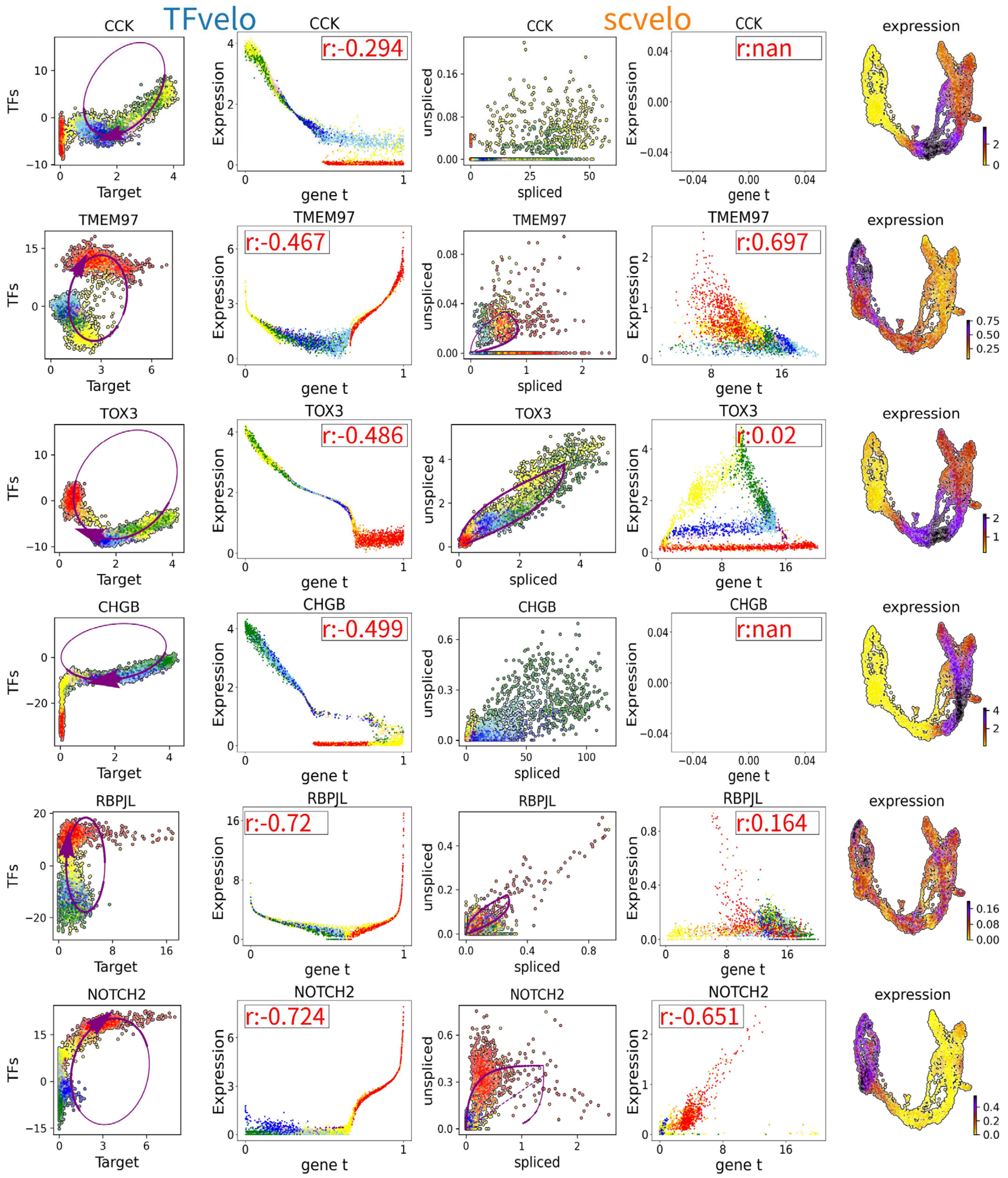

Figure S11. The comparison between TFvelo and scvelo on the genes selected from the pancreas study, including the dynamics fitting in phase portrait (1st and 3rd columns for TFvelo and scvelo respectively), and the expression along gene-specific latent time (2nd and 4th columns for TFvelo and scvelo respectively). The spearman correlation between each gene-specific time with Palantir pseudotime is shown in the plot.

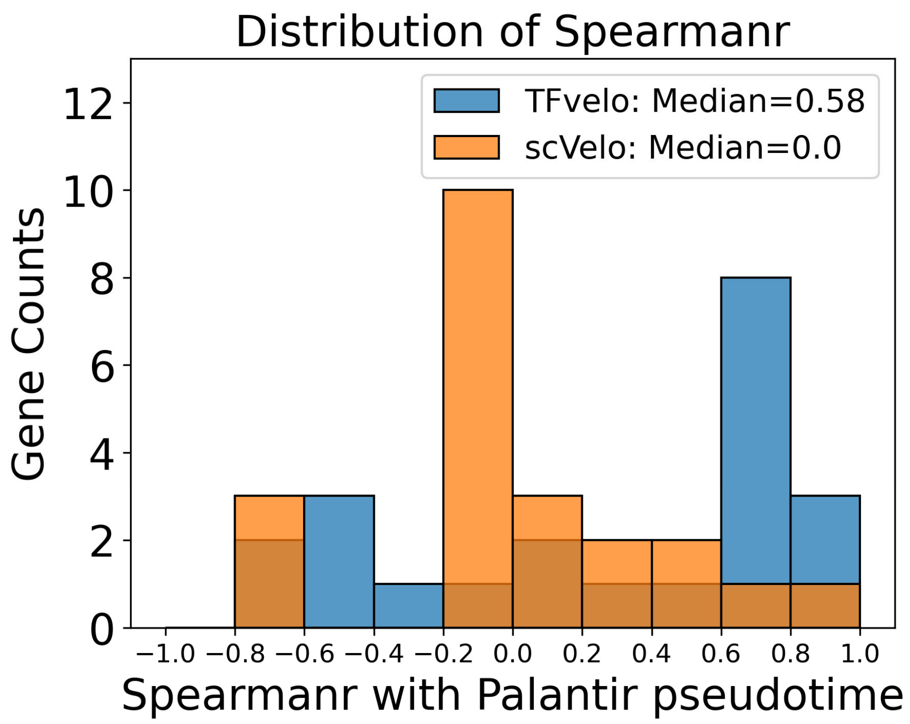

Figure S12. The comparison between TFvelo and scvelo, in terms of the distribution of Spearman correlation between the gene-specific latent time and the Palantir pseudotime, on functional genes selected from the pancreas study. The median value of Spearman correlations obtained by each method is shown in the figure.

After obtaining the pseudotime, an additional filtering step is adopted to select the best-fitted gene based on the models that are also consistent with pseudotime inference. Some genes may present more challenges for model fitting due to factors such as noise, sparsity, etc. We observe that there are still some genes that do not align with the correct results (**Fig. S14**). TFvelo aims to model dynamics on each gene more accurately and then extract cell-specific time based on the correctly fitted genes. To make the final stream-plot more robust and capturing the main dynamic processes described in the data, we introduce an additional filtering step to ensure that the models for all genes remain consistent with each other.

To illustrate the Robustness of TFvelo with this posterior processing strategy, we show the performance under different number of selected genes. Using the top-10, top-30, top-50, and top-100 genes where the gene-specific latent time best aligns with the pseudotime, we can always arrive at the similar results on stream plot **Fig. S13**. This is because genes selected in this way are fitted well and consistently.

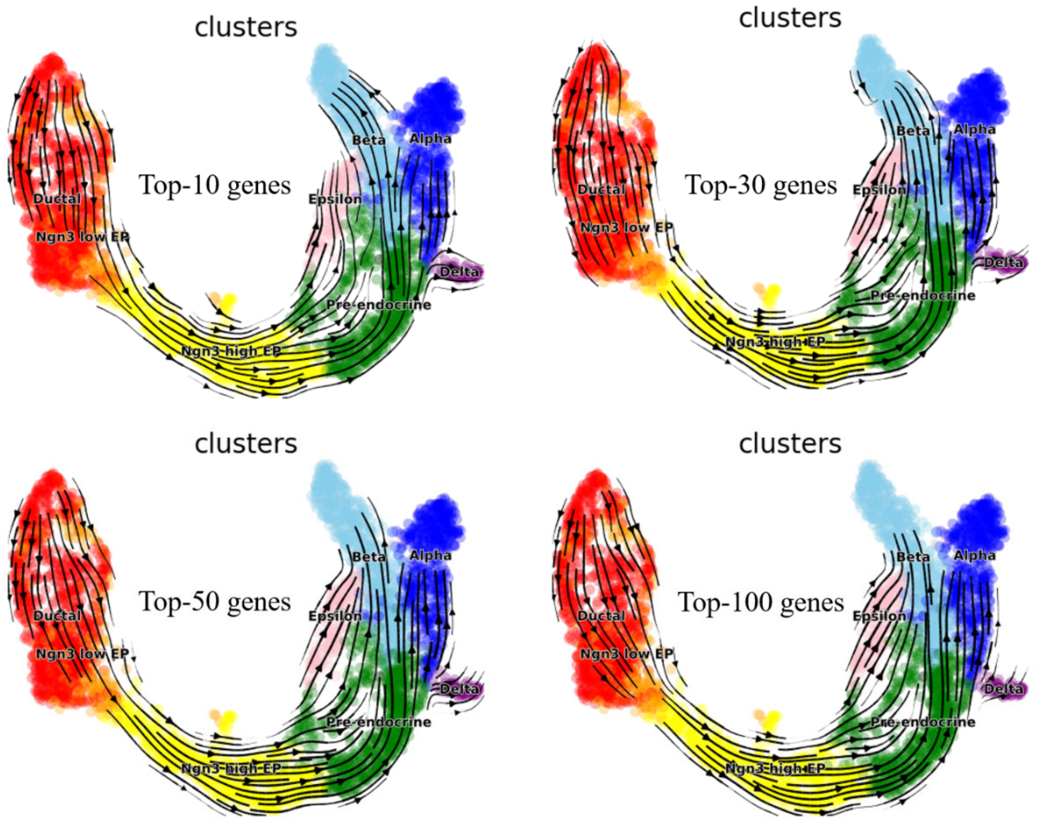

Figure S13. The stream-plot obtained from different number of velocity genes.

Some typical failure cases are shown in **Fig. S14**. Specifically, for some genes with low expression levels across most cell types, cells exhibiting low expression tend to be positioned along the TFs-axis in phase portraits, making their fitting challenging (**Fig. S14a**). Additionally, some genes exhibit a reversed order in latent time modeling (**Fig. S14b**). Moreover, TFvelo may encounter difficulties in effectively modeling some genes due to high noise, resulting in unsatisfactory fits (**Fig. S14c**).

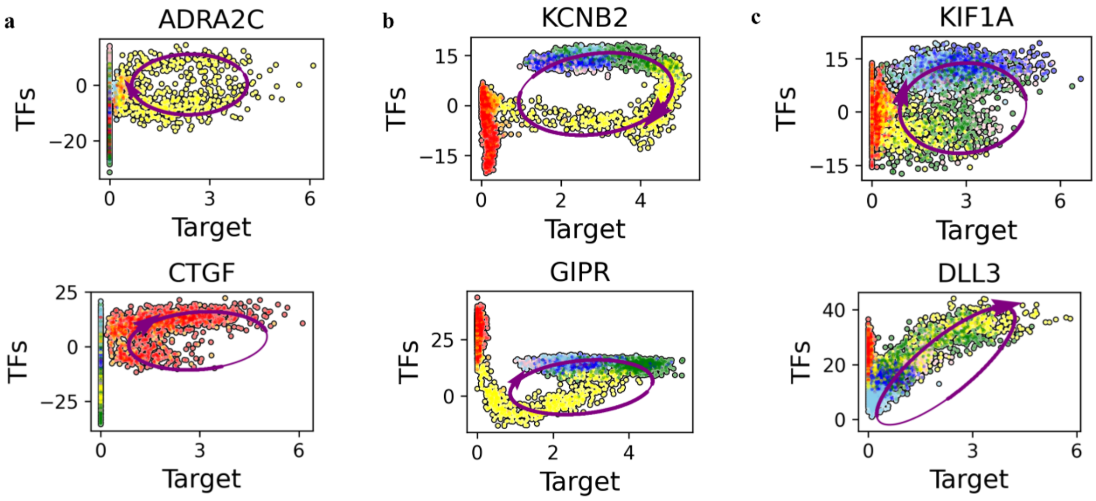

Figure S14. The failure case of TFvelo on modeling individual genes. (a) TFvelo

cannot fit the data when the gene is expressed in only one cell type. (b)TFvelo infers inverse dynamics. (c) TFvelo is affected by high noise.

5. Additional results on gastrulation erythroid dataset.

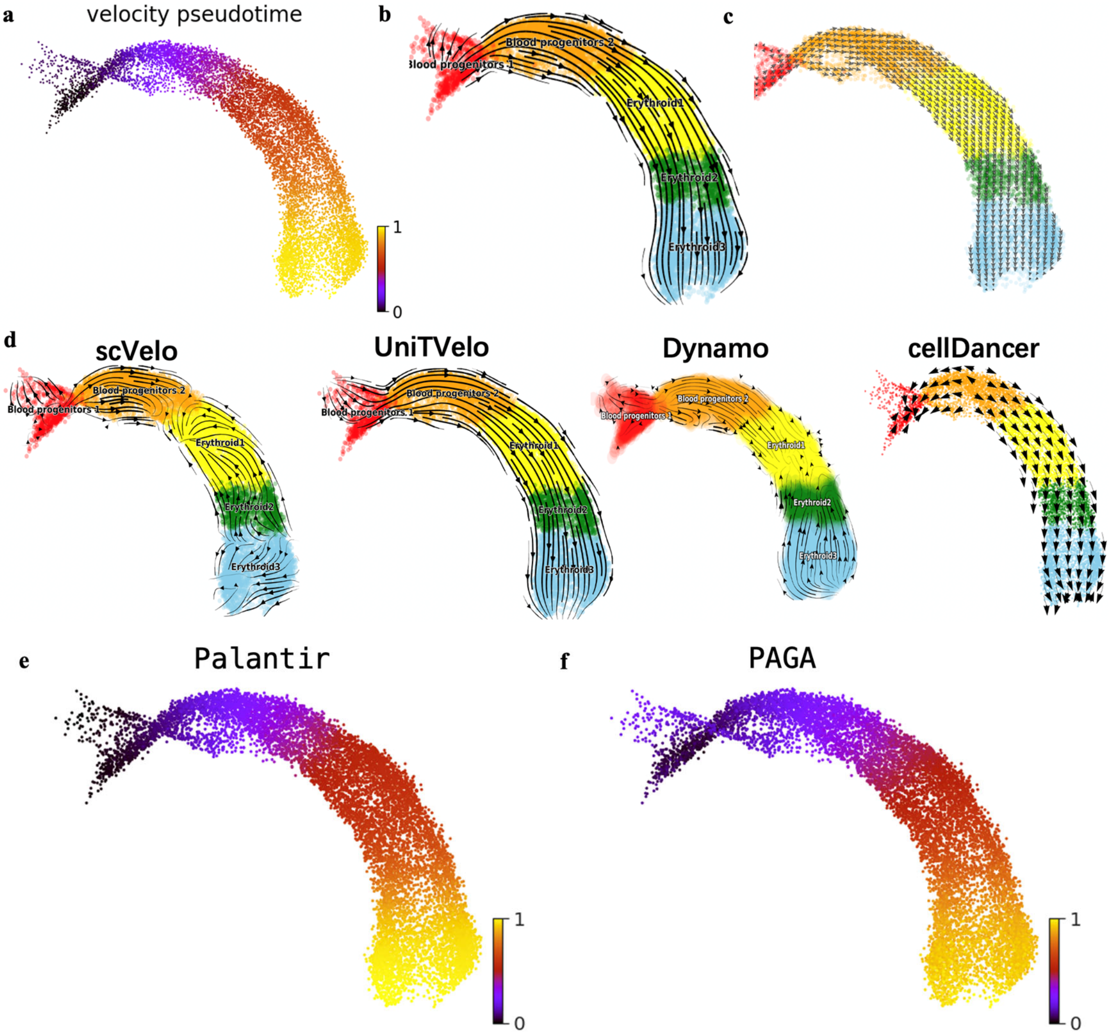

Figure S15. Comparison between TFvelo with baseline approaches on gastrulation erythroid dataset. (a) Pseudotime inferred by TFvelo on UMAP space. (b) Stream plot of TFvelo on UMAP space. (c) Grid plot of TFvelo on UMAP space. (d) Stream plot of baseline RNA velocity approaches on UMAP space, where the results are obtained by run the pipeline of them. (e) Pseudotime inferred by Palantir on UMAP space. (f) Pseudotime inferred by PAGA on UMAP space.

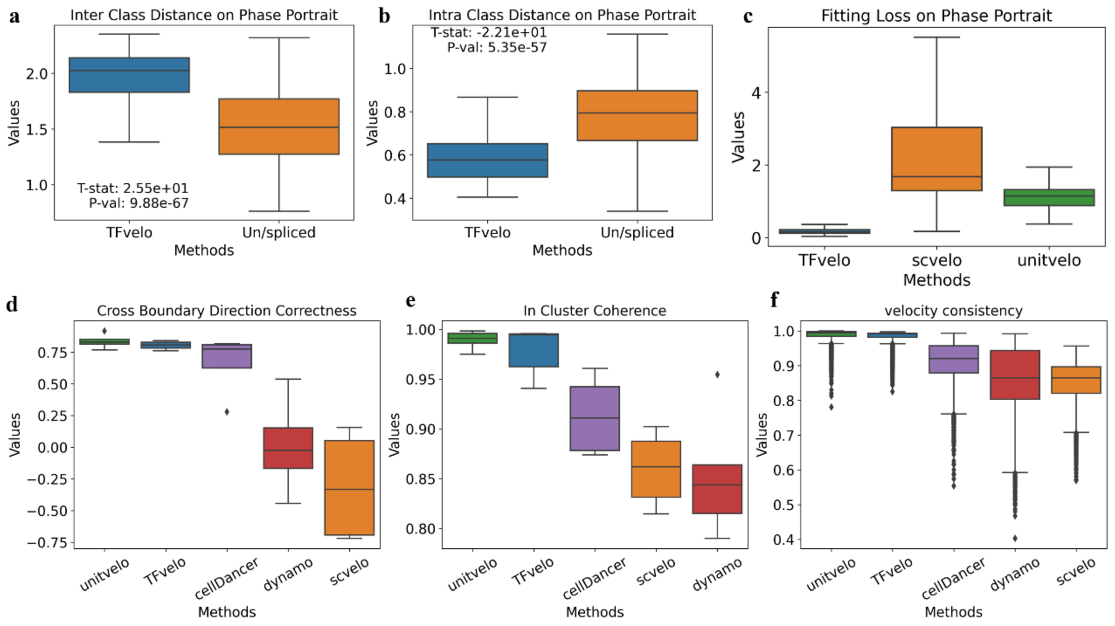

Figure S16. Quantitative comparison between TFvelo with baseline approaches on gastrulation erythroid dataset. (a-c) The quantitative comparisons in phase portrait fitting. (a) Intra class distance. Two-sided t-test is applied without adjustment. (b) Inter class distance. Two-sided t-test is applied without adjustment. (c) Fitting loss. (d-f) The quantitative comparisons of the velocity stream. (d) Cross boundary direction correctness. (e) In cluster coherence. (f) Velocity confidence. Source data are provided in the Supplementary Data file.

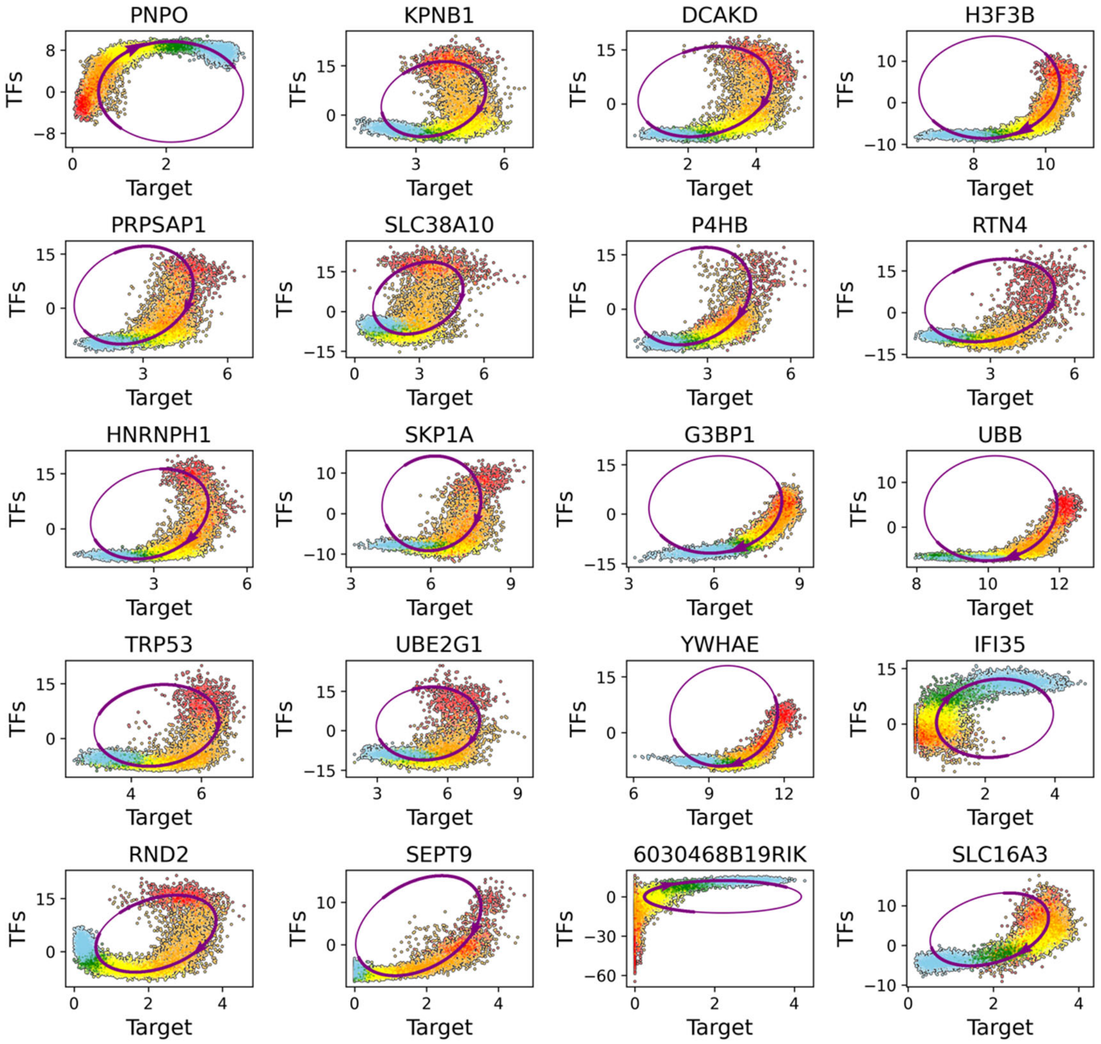

Figure S17. The phase portrait fitting of 20 example genes from gastrulation erythroid dataset. Cells are colored in the same way as **Fig. S5b**.

6. The root and end cells detection.

The same with scVelo, the root/end cells and velocity pseudotime is inferred based on the learned directed transition matrix. The root and end cells are derived from the stationary states of the Markov chains, represented by the velocity-inferred directed transition matrix and its transpose, respectively. This process involves identifying the 10 eigenvectors with the largest eigenvalues using the scipy.sparse.linalg.eigs() function. After that, among the 10 selected eigenvectors, those corresponding to eigenvalues lower than 0.999 are further filtered out. Subsequently, using the left eigenvectors and the connectivity graph between cells, which is constructed during preprocessing, we can get the distribution of root/end cells.

After smoothing the probability of root/end cell with the connectivity graph, the cell with the highest probability will be utilized as the root/end cell in pseudotime inference. Pseudotime for each cell is computed as the mean value of: (a) The number of steps it takes to reach the cell after starting to walk from the root cell, normalized to a range between zero and one, and (b) One minus the normalized number of steps it takes to reach the end point after starting to walk from the cell.

7. All TFs list

The TFs list is provided by the *Cell* paper: The Human Transcription Factors with PMID of 29425488, which includes 1639 TFs.

AC008770.3, AC023509.3, AC092835.1, AC138696.1, ADNP, ADNP2, AEBP1, AEBP2, AHCTF1, AHDC1, AHR, AHRR, AIRE, AKAP8, AKAP8L, AKNA, ALX1, ALX3, ALX4, ANHX, ANKZF1, AR, ARGFX, ARHGAP35, ARID2, ARID3A, ARID3B, ARID3C, ARID5A, ARID5B, ARNT, ARNT2, ARNTL, ARNTL2, ARX, ASCL1, ASCL2, ASCL3, ASCL4, ASCL5, ASH1L, ATF1, ATF2, ATF3, ATF4, ATF5, ATF6, ATF6B, ATF7, ATMIN, ATOH1, ATOH7, ATOH8, BACH1, BACH2, BARHL1, BARHL2, BARX1, BARX2, BATF, BATF2, BATF3, BAZ2A, BAZ2B, BBX, BCL11A, BCL11B, BCL6, BCL6B, BHLHA15, BHLHA9, BHLHE22, BHLHE23, BHLHE40, BHLHE41, BNC1, BNC2, BORCS8-MEF2B, BPTF, BRF2, BSX, C11orf95, CAMTA1, CAMTA2, CARF, CASZ1, CBX2, CC2D1A, CCDC169-SOHLH2, CCDC17, CDC5L, CDX1, CDX2, CDX4, CEBPA, CEBPB, CEBPD, CEBPE, CEBPG, CEBPZ, CENPA, CENPB, CENPBD1, CENPS, CENPT, CENPX, CGGBP1, CHAMP1, CHCHD3, CIC, CLOCK, CPEB1, CPXCR1, CREB1, CREB3, CREB3L1, CREB3L2, CREB3L3, CREB3L4, CREB5, CREBL2, CREBZF, CREM, CRX, CSRNP1, CSRNP2, CSRNP3, CTCF, CTCFL, CUX1, CUX2, CXXC1, CXXC4, CXXC5, DACH1, DACH2, DBP, DBX1, DBX2, DDIT3, DEAF1, DLX1, DLX2, DLX3, DLX4, DLX5, DLX6, DMBX1, DMRT1, DMRT2, DMRT3, DMRTA1, DMRTA2, DMRTB1, DMRTC2, DMTF1, DNMT1, DNTTIP1, DOT1L, DPF1, DPF3, DPRX, DR1, DRAP1, DRGX, DUX1, DUX3, DUX4, DUXA, DZIP1, E2F1, E2F2, E2F3, E2F4, E2F5, E2F6, E2F7, E2F8, E4F1, EBF1, EBF2, EBF3, EBF4, EEA1, EGR1, EGR2, EGR3, EGR4, EHF, ELF1, ELF2, ELF3, ELF4, ELF5, ELK1, ELK3, ELK4, EMX1, EMX2, EN1, EN2, EOMES, EPAS1, ERF, ERG, ESR1, ESR2, ESRRA, ESRRB, ESRRG, ESX1, ETS1, ETS2, ETV1, ETV2, ETV3, ETV3L, ETV4, ETV5, ETV6, ETV7, EVX1, EVX2, FAM170A, FAM200B, FBXL19, FERD3L, FEV, FEZF1, FEZF2, FIGLA, FIZ1, FLI1, FLYWCH1, FOS, FOSB, FOSL1, FOSL2, FOXA1, FOXA2, FOXA3, FOXB1, FOXB2, FOXC1, FOXC2, FOXD1, FOXD2, FOXD3, FOXD4, FOXD4L1, FOXD4L3, FOXD4L4, FOXD4L5, FOXD4L6, FOXE1, FOXE3, FOXF1, FOXF2, FOXG1, FOXH1, FOXI1, FOXI2, FOXI3, FOXJ1, FOXJ2, FOXJ3, FOXK1, FOXK2, FOXL1, FOXL2, FOXM1, FOXN1, FOXN2, FOXN3, FOXN4, FOXO1, FOXO3, FOXO4, FOXO6, FOXP1, FOXP2, FOXP3, FOXP4, FOXQ1, FOXR1, FOXR2, FOXS1, GABPA, GATA1, GATA2, GATA3, GATA4, GATA5, GATA6, GATAD2A, GATAD2B, GBX1, GBX2, GCM1, GCM2, GFI1, GFI1B, GLI1, GLI2, GLI3, GLI4, GLIS1, GLIS2, GLIS3, GLMP, GLYR1, GMEB1, GMEB2, GPBP1, GPBP1L1, GRHL1, GRHL2, GRHL3, GSC, GSC2, GSX1, GSX2, GTF2B, GTF2I, GTF2IRD1, GTF2IRD2, GTF2IRD2B, GTF3A, GZF1, HAND1, HAND2, HBP1, HDX, HELT, HES1, HES2, HES3, HES4, HES5, HES6, HES7, HESX1, HEY1, HEY2, HEYL, HHEX, HIC1, HIC2, HIF1A, HIF3A, HINFP, HIVEP1, HIVEP2, HIVEP3, HKR1, HLF, HLX, HMBOX1, HMG20A, HMG20B, HMGA1, HMGA2, HMGN3, HMX1, HMX2, HMX3, HNF1A, HNF1B, HNF4A, HNF4G, HOMEZ,

HOXA1, HOXA10, HOXA11, HOXA13, HOXA2, HOXA3, HOXA4, HOXA5, HOXA6, HOXA7, HOXA9, HOXB1, HOXB13, HOXB2, HOXB3, HOXB4, HOXB5, HOXB6, HOXB7, HOXB8, HOXB9, HOXC10, HOXC11, HOXC12, HOXC13, HOXC4, HOXC5, HOXC6, HOXC8, HOXC9, HOXD1, HOXD10, HOXD11, HOXD12, HOXD13, HOXD3, HOXD4, HOXD8, HOXD9, HSF1, HSF2, HSF4, HSF5, HSFX1, HSFX2, HSFY1, HSFY2, IKZF1, IKZF2, IKZF3, IKZF4, IKZF5, INSM1, INSM2, IRF1, IRF2, IRF3, IRF4, IRF5, IRF6, IRF7, IRF8, IRF9, IRX1, IRX2, IRX3, IRX4, IRX5, IRX6, ISL1, ISL2, ISX, JAZF1, JDP2, JRK, JRKL, JUN, JUNB, JUND, KAT7, KCMF1, KCNIP3, KDM2A, KDM2B, KDM5B, KIN, KLF1, KLF10, KLF11, KLF12, KLF13, KLF14, KLF15, KLF16, KLF17, KLF2, KLF3, KLF4, KLF5, KLF6, KLF7, KLF8, KLF9, KMT2A, KMT2B, L3MBTL1, L3MBTL3, L3MBTL4, LBX1, LBX2, LCOR, LCORL, LEF1, LEUTX, LHX1, LHX2, LHX3, LHX4, LHX5, LHX6, LHX8, LHX9, LIN28A, LIN28B, LIN54, LMX1A, LMX1B, LTF, LYL1, MAF, MAFA, MAFB, MAFF, MAFG, MAFK, MAX, MAZ, MBD1, MBD2, MBD3, MBD4, MBD6, MBNL2, MECOM, MECP2, MEF2A, MEF2B, MEF2C, MEF2D, MEIS1, MEIS2, MEIS3, MEOX1, MEOX2, MESP1, MESP2, MGA, MITF, MIXL1, MKX, MLX, MLXIP, MLXIPL, MNT, MNX1, MSANTD1, MSANTD3, MSANTD4, MSC, MSGN1, MSX1, MSX2, MTERF1, MTERF2, MTERF3, MTERF4, MTF1, MTF2, MXD1, MXD3, MXD4, MXI1, MYB, MYBL1, MYBL2, MYC, MYCL, MYCN, MYF5, MYF6, MYNN, MYOD1, MYOG, MYPOP, MYRF, MYRFL, MYSM1, MYT1, MYT1L, MZF1, NACC2, NAIF1, NANOG, NANOGNB, NANOGP8, NCOA1, NCOA2, NCOA3, NEUROD1, NEUROD2, NEUROD4, NEUROD6, NEUROG1, NEUROG2, NEUROG3, NFAT5, NFATC1, NFATC2, NFATC3, NFATC4, NFE2, NFE2L1, NFE2L2, NFE2L3, NFE4, NFIA, NFIB, NFIC, NFIL3, NFIX, NFKB1, NFKB2, NFX1, NFXL1, NFYA, NFYB, NFYC, NHLH1, NHLH2, NKRF, NKX1-1, NKX1-2, NKX2-1, NKX2-2, NKX2-3, NKX2-4, NKX2-5, NKX2-6, NKX2-8, NKX3-1, NKX3-2, NKX6-1, NKX6-2, NKX6-3, NME2, NOBOX, NOTO, NPAS1, NPAS2, NPAS3, NPAS4, NR0B1, NR1D1, NR1D2, NR1H2, NR1H3, NR1H4, NR1I2, NR1I3, NR2C1, NR2C2, NR2E1, NR2E3, NR2F1, NR2F2, NR2F6, NR3C1, NR3C2, NR4A1, NR4A2, NR4A3, NR5A1, NR5A2, NR6A1, NRF1, NRL, OLIG1, OLIG2, OLIG3, ONECUT1, ONECUT2, ONECUT3, OSR1, OSR2, OTP, OTX1, OTX2, OVOL1, OVOL2, OVOL3, PA2G4, PATZ1, PAX1, PAX2, PAX3, PAX4, PAX5, PAX6, PAX7, PAX8, PAX9, PBX1, PBX2, PBX3, PBX4, PCGF2, PCGF6, PDX1, PEG3, PGR, PHF1, PHF19 , PHF20, PHF21A, PHOX2A, PHOX2B, PIN1, PITX1, PITX2, PITX3, PKNOX1, PKNOX2, PLAG1, PLAGL1, PLAGL2, PLSCR1, POGK, POU1F1, POU2AF1, POU2F1, POU2F2, POU2F3, POU3F1, POU3F2, POU3F3, POU3F4, POU4F1, POU4F2, POU4F3, POU5F1, POU5F1B, POU5F2, POU6F1, POU6F2, PPARA, PPARD, PPARG, PRDM1, PRDM10, PRDM12, PRDM13, PRDM14, PRDM15, PRDM16, PRDM2, PRDM4, PRDM5, PRDM6, PRDM8, PRDM9, PREB, PRMT3, PROP1, PROX1, PROX2, PRR12, PRRX1, PRRX2, PTF1A, PURA, PURB, PURG, RAG1, RARA, RARB, RARG, RAX, RAX2, RBAK, RBCK1, RBPJ, RBPJL, RBSN, REL, RELA, RELB, REPIN1, REST, REXO4, RFX1, RFX2, RFX3, RFX4, RFX5, RFX6, RFX7, RFX8, RHOXF1, RHOXF2, RHOXF2B, RLF, RORA, RORB, RORC, RREB1, RUNX1, RUNX2, RUNX3, RXRA, RXRB, RXRG, SAFB, SAFB2,

SALL1, SALL2, SALL3, SALL4, SATB1, SATB2, SCMH1, SCML4, SCRT1, SCRT2, SCX, SEBOX, SETBP1, SETDB1, SETDB2, SGSM2, SHOX, SHOX2, SIM1, SIM2, SIX1, SIX2, SIX3, SIX4, SIX5, SIX6, SKI, SKIL, SKOR1, SKOR2, SLC2A4RG, SMAD1, SMAD3, SMAD4, SMAD5, SMAD9, SMYD3, SNAI1, SNAI2, SNAI3, SNAPC2, SNAPC4, SNAPC5, SOHLH1, SOHLH2, SON, SOX1, SOX10, SOX11, SOX12, SOX13, SOX14, SOX15, SOX17, SOX18, SOX2, SOX21, SOX3, SOX30, SOX4, SOX5, SOX6, SOX7, SOX8, SOX9, SP1, SP100, SP110, SP140, SP140L, SP2, SP3, SP4, SP5, SP6, SP7, SP8, SP9, SPDEF, SPEN, SPI1, SPIB, SPIC, SPZ1, SRCAP, SREBF1, SREBF2, SRF, SRY, ST18, STAT1, STAT2, STAT3, STAT4, STAT5A, STAT5B, STAT6, T, TAL1, TAL2, TBP, TBPL1, TBPL2, TBR1, TBX1, TBX10, TBX15, TBX18, TBX19, TBX2, TBX20, TBX21, TBX22, TBX3, TBX4, TBX5, TBX6, TCF12, TCF15, TCF20, TCF21, TCF23, TCF24, TCF3, TCF4, TCF7, TCF7L1, TCF7L2, TCFL5, TEAD1, TEAD2, TEAD3, TEAD4, TEF, TERB1, TERF1, TERF2, TET1, TET2, TET3, TFAP2A, TFAP2B, TFAP2C, TFAP2D, TFAP2E, TFAP4, TFCP2, TFCP2L1, TFDP1, TFDP2, TFDP3, TFE3, TFEB, TFEC, TGIF1, TGIF2, TGIF2LX, TGIF2LY, THAP1, THAP10, THAP11, THAP12, THAP2, THAP3, THAP4, THAP5, THAP6, THAP7, THAP8, THAP9, THRA, THRB, THYN1, TIGD1, TIGD2, TIGD3, TIGD4, TIGD5, TIGD6, TIGD7, TLX1, TLX2, TLX3, TMF1, TOPORS, TP53, TP63, TP73, TPRX1, TRAFD1, TRERF1, TRPS1, TSC22D1, TSHZ1, TSHZ2, TSHZ3, TTF1, TWIST1, TWIST2, UBP1, UNCX, USF1, USF2, USF3, VAX1, VAX2, VDR, VENTX, VEZF1, VSX1, VSX2, WIZ, WT1, XBP1, XPA, YBX1, YBX2, YBX3, YY1, YY2, ZBED1, ZBED2, ZBED3, ZBED4, ZBED5, ZBED6, ZBED9, ZBTB1, ZBTB10, ZBTB11, ZBTB12, ZBTB14, ZBTB16, ZBTB17, ZBTB18, ZBTB2, ZBTB20, ZBTB21, ZBTB22, ZBTB24, ZBTB25, ZBTB26, ZBTB3, ZBTB32, ZBTB33, ZBTB34, ZBTB37, ZBTB38, ZBTB39, ZBTB4, ZBTB40, ZBTB41, ZBTB42, ZBTB43, ZBTB44, ZBTB45, ZBTB46, ZBTB47, ZBTB48, ZBTB49, ZBTB5, ZBTB6, ZBTB7A, ZBTB7B, ZBTB7C, ZBTB8A, ZBTB8B, ZBTB9, ZC3H8, ZEB1, ZEB2, ZFAT, ZFHX2, ZFHX3, ZFHX4, ZFP1, ZFP14, ZFP2, ZFP28, ZFP3, ZFP30, ZFP37, ZFP41, ZFP42, ZFP57, ZFP62, ZFP64, ZFP69, ZFP69B, ZFP82, ZFP90, ZFP91, ZFP92, ZFPM1, ZFPM2, ZFX, ZFY, ZGLP1, ZGPAT, ZHX1, ZHX2, ZHX3, ZIC1, ZIC2, ZIC3, ZIC4, ZIC5, ZIK1, ZIM2, ZIM3, ZKSCAN1, ZKSCAN2, ZKSCAN3, ZKSCAN4, ZKSCAN5, ZKSCAN7, ZKSCAN8, ZMAT1, ZMAT4, ZNF10, ZNF100, ZNF101, ZNF107, ZNF112, ZNF114, ZNF117, ZNF12, ZNF121, ZNF124, ZNF131, ZNF132, ZNF133, ZNF134, ZNF135, ZNF136, ZNF138, ZNF14, ZNF140, ZNF141, ZNF142, ZNF143, ZNF146, ZNF148, ZNF154, ZNF155, ZNF157, ZNF16, ZNF160, ZNF165, ZNF169, ZNF17, ZNF174, ZNF175, ZNF177, ZNF18, ZNF180, ZNF181, ZNF182, ZNF184, ZNF189, ZNF19, ZNF195, ZNF197, ZNF2, ZNF20, ZNF200, ZNF202, ZNF205, ZNF207, ZNF208, ZNF211, ZNF212, ZNF213, ZNF214, ZNF215, ZNF217, ZNF219, ZNF22, ZNF221, ZNF222, ZNF223, ZNF224, ZNF225, ZNF226, ZNF227, ZNF229, ZNF23, ZNF230, ZNF232, ZNF233, ZNF234, ZNF235, ZNF236, ZNF239, ZNF24, ZNF248, ZNF25, ZNF250, ZNF251, ZNF253, ZNF254, ZNF256, ZNF257, ZNF26, ZNF260, ZNF263, ZNF264, ZNF266, ZNF267, ZNF268, ZNF273, ZNF274, ZNF275, ZNF276, ZNF277, ZNF28, ZNF280A, ZNF280B, ZNF280C, ZNF280D, ZNF281, ZNF282, ZNF283, ZNF284, ZNF285, ZNF286A, ZNF286B,

ZNF287, ZNF292, ZNF296, ZNF3, ZNF30, ZNF300, ZNF302, ZNF304, ZNF311, ZNF316, ZNF317, ZNF318, ZNF319, ZNF32, ZNF320, ZNF322, ZNF324, ZNF324B, ZNF326, ZNF329, ZNF331, ZNF333, ZNF334, ZNF335, ZNF337, ZNF33A, ZNF33B, ZNF34, ZNF341, ZNF343, ZNF345, ZNF346, ZNF347, ZNF35, ZNF350, ZNF354A, ZNF354B, ZNF354C, ZNF358, ZNF362, ZNF365, ZNF366, ZNF367, ZNF37A, ZNF382, ZNF383, ZNF384, ZNF385A, ZNF385B, ZNF385C, ZNF385D, ZNF391, ZNF394, ZNF395, ZNF396, ZNF397, ZNF398, ZNF404, ZNF407, ZNF408, ZNF41, ZNF410, ZNF414, ZNF415, ZNF416, ZNF417, ZNF418, ZNF419, ZNF420, ZNF423, ZNF425, ZNF426, ZNF428, ZNF429, ZNF43, ZNF430, ZNF431, ZNF432, ZNF433, ZNF436, ZNF438, ZNF439, ZNF44, ZNF440, ZNF441, ZNF442, ZNF443, ZNF444, ZNF445, ZNF446, ZNF449, ZNF45, ZNF451, ZNF454, ZNF460, ZNF461, ZNF462, ZNF467, ZNF468, ZNF469, ZNF470, ZNF471, ZNF473, ZNF474, ZNF479, ZNF48, ZNF480, ZNF483, ZNF484, ZNF485, ZNF486, ZNF487, ZNF488, ZNF490, ZNF491, ZNF492, ZNF493, ZNF496, ZNF497, ZNF500, ZNF501, ZNF502, ZNF503, ZNF506, ZNF507, ZNF510, ZNF511, ZNF512, ZNF512B, ZNF513, ZNF514, ZNF516, ZNF517, ZNF518A, ZNF518B, ZNF519, ZNF521, ZNF524, ZNF525, ZNF526, ZNF527, ZNF528, ZNF529, ZNF530, ZNF532, ZNF534, ZNF536, ZNF540, ZNF541, ZNF543, ZNF544, ZNF546, ZNF547, ZNF548, ZNF549, ZNF550, ZNF551, ZNF552, ZNF554, ZNF555, ZNF556, ZNF557, ZNF558, ZNF559, ZNF560, ZNF561, ZNF562, ZNF563, ZNF564, ZNF565, ZNF566, ZNF567, ZNF568, ZNF569, ZNF57, ZNF570, ZNF571, ZNF572, ZNF573, ZNF574, ZNF575, ZNF576, ZNF577, ZNF578, ZNF579, ZNF580, ZNF581, ZNF582, ZNF583, ZNF584, ZNF585A, ZNF585B, ZNF586, ZNF587, ZNF587B, ZNF589, ZNF592, ZNF594, ZNF595, ZNF596, ZNF597, ZNF598, ZNF599, ZNF600, ZNF605, ZNF606, ZNF607, ZNF608, ZNF609, ZNF610, ZNF611, ZNF613, ZNF614, ZNF615, ZNF616, ZNF618, ZNF619, ZNF620, ZNF621, ZNF623, ZNF624, ZNF625, ZNF626, ZNF627, ZNF628, ZNF629, ZNF630, ZNF639, ZNF641, ZNF644, ZNF645, ZNF646, ZNF648, ZNF649, ZNF652, ZNF653, ZNF654, ZNF655, ZNF658, ZNF66, ZNF660, ZNF662, ZNF664, ZNF665, ZNF667, ZNF668, ZNF669, ZNF670, ZNF671, ZNF672, ZNF674, ZNF675, ZNF676, ZNF677, ZNF678, ZNF679, ZNF680, ZNF681, ZNF682, ZNF683, ZNF684, ZNF687, ZNF688, ZNF689, ZNF69, ZNF691, ZNF692, ZNF695, ZNF696, ZNF697, ZNF699, ZNF7, ZNF70, ZNF700, ZNF701, ZNF703, ZNF704, ZNF705A, ZNF705B, ZNF705D, ZNF705E, ZNF705G, ZNF706, ZNF707, ZNF708, ZNF709, ZNF71, ZNF710, ZNF711, ZNF713, ZNF714, ZNF716, ZNF717, ZNF718, ZNF721, ZNF724, ZNF726, ZNF727, ZNF728, ZNF729, ZNF730, ZNF732, ZNF735, ZNF736, ZNF737, ZNF74, ZNF740, ZNF746, ZNF747, ZNF749, ZNF750, ZNF75A, ZNF75D, ZNF76, ZNF761, ZNF763, ZNF764, ZNF765, ZNF766, ZNF768, ZNF77, ZNF770, ZNF771, ZNF772, ZNF773, ZNF774, ZNF775, ZNF776, ZNF777, ZNF778, ZNF780A, ZNF780B, ZNF781, ZNF782, ZNF783, ZNF784, ZNF785, ZNF786, ZNF787, ZNF788, ZNF789, ZNF79, ZNF790, ZNF791, ZNF792, ZNF793, ZNF799, ZNF8, ZNF80, ZNF800, ZNF804A, ZNF804B, ZNF805, ZNF808, ZNF81, ZNF813, ZNF814, ZNF816, ZNF821, ZNF823, ZNF827, ZNF829, ZNF83, ZNF830, ZNF831, ZNF835, ZNF836, ZNF837, ZNF84, ZNF841, ZNF843, ZNF844, ZNF845, ZNF846, ZNF85, ZNF850, ZNF852, ZNF853, ZNF860, ZNF865, ZNF878, ZNF879, ZNF880, ZNF883, ZNF888, ZNF891, ZNF90, ZNF91,

ZNF92, ZNF93, ZNF98, ZNF99, ZSCAN1, ZSCAN10, ZSCAN12, ZSCAN16, ZSCAN18, ZSCAN2, ZSCAN20, ZSCAN21, ZSCAN22, ZSCAN23, ZSCAN25, ZSCAN26, ZSCAN29, ZSCAN30, ZSCAN31, ZSCAN32, ZSCAN4, ZSCAN5A, ZSCAN5B, ZSCAN5C, ZSCAN9, ZUFSP, ZXDA, ZXDB, ZXDC, ZZZ3

8. Supplementary References

1 Lambert, S. A. *et al.* The human transcription factors. *Cell* **172**, 650-665 (2018).

2 La Manno, G. *et al.* RNA velocity of single cells. *Nature* **560**, 494-498 (2018).

3 Bastidas-Ponce, A. *et al.* Comprehensive single cell mRNA profiling reveals a detailed roadmap for pancreatic endocrinogenesis. *Development* **146**, dev173849 (2019).
