# Supplementary material for: TFvelo: gene regulation inspired RNA velocity estimation
Source: Nat Commun. 2024 Feb 15;15:1387. doi: 10.1038/s41467-024-45661-w (PMC11258302; doi:10.1038/s41467-024-45661-w)
Supplement: Supplementary file 3 — Reporting Summary [file 41467_2024_45661_MOESM3_ESM.pdf]

nature portfolio

Corresponding author(s): Ye Yuan and Hong-bin Shen

Last updated by author(s): Jan 10, 2024

Reporting Summary

Nature Portfolio wishes to improve the reproducibility of the work that we publish. This form provides structure for consistency and transparency in reporting. For further information on Nature Portfolio policies, see our Editorial Policies and the Editorial Policy Checklist.

Statistics

For all statistical analyses, confirm that the following items are present in the figure legend, table legend, main text, or Methods section.

n/a	Confirmed	
☐	☒	The exact sample size (*n*) for each experimental group/condition, given as a discrete number and unit of measurement
☐	☒	A statement on whether measurements were taken from distinct samples or whether the same sample was measured repeatedly
☐	☒	The statistical test(s) used AND whether they are one- or two-sided<br>*Only common tests should be described solely by name; describe more complex techniques in the Methods section.*
☐	☒	A description of all covariates tested
☐	☒	A description of any assumptions or corrections, such as tests of normality and adjustment for multiple comparisons
☐	☒	A full description of the statistical parameters including central tendency (e.g. means) or other basic estimates (e.g. regression coefficient) AND variation (e.g. standard deviation) or associated estimates of uncertainty (e.g. confidence intervals)
☐	☒	For null hypothesis testing, the test statistic (e.g. *F*, *t*, *r*) with confidence intervals, effect sizes, degrees of freedom and *P* value noted<br>*Give P values as exact values whenever suitable.*
☐	☒	For Bayesian analysis, information on the choice of priors and Markov chain Monte Carlo settings
☐	☒	For hierarchical and complex designs, identification of the appropriate level for tests and full reporting of outcomes
☐	☒	Estimates of effect sizes (e.g. Cohen's *d*, Pearson's *r*), indicating how they were calculated

Our web collection on statistics for biologists contains articles on many of the points above.

Software and code

Policy information about availability of computer code

Data collection	No software was used for data collection, please state here accordingly.
Data analysis	TFvelo is implemented in Python, based on the scVelo package. The source code can be downloaded from the GitHub repository, https://github.com/xiaoyeye/TFvelo.

For manuscripts utilizing custom algorithms or software that are central to the research but not yet described in published literature, software must be made available to editors and reviewers. We strongly encourage code deposition in a community repository (e.g. GitHub). See the Nature Portfolio guidelines for submitting code & software for further information.

Data

Policy information about availability of data

All manuscripts must include a data availability statement. This statement should provide the following information, where applicable:

- Accession codes, unique identifiers, or web links for publicly available datasets
- A description of any restrictions on data availability
- For clinical datasets or third party data, please ensure that the statement adheres to our policy

The pancreatic endocrinogenesis dataset 59 is available from NCBI GEO under accession ID GSE132188. It comprises the single-cell RNA-seq (10X) data of 27,998 genes of 3,696 pancreatic epithelial and Ngn3-Venus fusion cells sampled from mouse embryonic day 15.5. Data could be acquired from scvelo.datasets.pancreas().

The gastrulation erythroid dataset, which is selected from the transcriptional profiles of mouse embryos 47, provides expressions of 53,801 genes of 9,815 cells. This dataset is incorporated by scvelo.datasets.gastrulation_erythroid().

10x embryonic mouse brain dataset can be accessed at the 10x website at https://www.10xgenomics.com/resources/datasets/fresh-embryonic-e-18-mouse-brain-5-k-1-standard-1-0-0. To ensure a fair comparison between TFvelo and Multivelo, TFvelo utilizes the same RNA data file with the one used in Multivelo (https://multivelo.readthedocs.io/en/latest/MultiVelo_Fig2.html), which consists of 3365 cells and 936 genes. The preprocessed data used in this study is provided at https://github.com/xiaoyeye/TFvelo/blob/main/data/10x_mouse_brain/adata_rna.h5ad.

Human preimplantation embryos dataset is a single-cell RNA-seq dataset of 1,529 cells obtained from 88 human preimplantation embryos ranging from embryonic day 3 to 7. Data were downloaded with the accession number of E-MTAB-3929 from EMBL-EBI 54. In this dataset, only the RNA abundance is provided. As a result, those RNA velocity methods relying on the spliced/unspliced are not available.

The ENCODE TF-target database is available at: https://maayanlab.cloud/Harmonizome/dataset/ENCODE+Transcription+Factor+Targets.
The ChEA TF-target database is available at: https://maayanlab.cloud/Harmonizome/dataset/CHEA+Transcription+Factor+Targets.

Research involving human participants, their data, or biological material

Policy information about studies with human participants or human data. See also policy information about sex, gender (identity/presentation), and sexual orientation and race, ethnicity and racism.

Reporting on sex and gender	No human research participants are involved in this study.
Reporting on race, ethnicity, or other socially relevant groupings	N/A
Population characteristics	N/A
Recruitment	N/A
Ethics oversight	N/A

Note that full information on the approval of the study protocol must also be provided in the manuscript.

Field-specific reporting

Please select the one below that is the best fit for your research. If you are not sure, read the appropriate sections before making your selection.

☒ Life sciences ☐ Behavioural & social sciences ☐ Ecological, evolutionary & environmental sciences

For a reference copy of the document with all sections, see nature.com/documents/nr-reporting-summary-flat.pdf

Life sciences study design

All studies must disclose on these points even when the disclosure is negative.

Sample size	We use four datasets in this study. Each of them includes 3696 (pancreas), 9815 (gastrulation erythroid), 3365 (10x embryonic mouse brain) and 1529 (human pre-implantation embryos) cells, respectively. Most relevant RNA velocity studies show results on 3 or 4 datasets in their papers. The pancreas, gastrulation erythroid and 10x embryonic mouse brain datasets have been utilized in these previous RNA velocity methods. So we determined to employ these datasets in this study.
Data exclusions	No data were excluded.
Replication	The results on these datasets can be reproduced by running the code at https://github.com/xiaoyeye/TFvelo.
Randomization	TFvelo is a computational tool for trajectory analysis on single cell data. This is an unsupervised learning task, where all samples (cells) are input to the model without being allocated into different groups. So randomization is not relevant to this study.
Blinding	TFvelo is a computational tool for trajectory analysis on single cell data. This is an unsupervised learning task, where all samples (cells) are input to the model without being allocated into different groups. So blinding is not relevant to this study.

Reporting for specific materials, systems and methods

We require information from authors about some types of materials, experimental systems and methods used in many studies. Here, indicate whether each material, system or method listed is relevant to your study. If you are not sure if a list item applies to your research, read the appropriate section before selecting a response.

Materials & experimental systems

n/a	Involved in the study	
☒	☐	Antibodies
☒	☐	Eukaryotic cell lines
☒	☐	Palaeontology and archaeology
☒	☐	Animals and other organisms
☒	☐	Clinical data
☒	☐	Dual use research of concern
☒	☐	Plants

Methods

n/a	Involved in the study	
☒	☐	ChIP-seq
☒	☐	Flow cytometry
☒	☐	MRI-based neuroimaging

Plants

Seed stocks	Not involved in the study
Novel plant genotypes	Not involved in the study
Authentication	Not involved in the study
